# Myeloperoxidase and Thyrotropin‐Releasing Hormone Within Leukaemia Stem Cells Increased Chemosensitivity in Acute Myeloid Leukaemia

**DOI:** 10.1111/jcmm.70306

**Published:** 2024-12-25

**Authors:** Chung‐Hsing Chen, Tsung‐Chih Chen, Ting‐Shuan Wu, Tzu‐Hung Hsiao, Jo‐Mei Maureen Chen, Chi‐Ying F. Huang, Po‐Liang Cheng, Jia‐Rung Tsai, Chieh‐Lin Jerry Teng

**Affiliations:** ^1^ Department of Mathematics University of Taipei Taipei Taiwan; ^2^ National Institute of Cancer Research National Health Research Institutes Zhunan Taiwan; ^3^ Division of Hematology/Medical Oncology, Department of Medicine Taichung Veterans General Hospital Taichung Taiwan; ^4^ Department of Post‐Baccalaureate Medicine, College of Medicine National Chung Hsing University Taichung Taiwan; ^5^ Department of Biomedical Sciences Chung Shan Medical University Taichung Taiwan; ^6^ Department of Medical Research Chung Shan Medical University Hospital Taichung Taiwan; ^7^ Department of Medical Research Taichung Veterans General Hospital Taichung Taiwan; ^8^ Department of Public Health Fu Jen Catholic University New Taipei City Taiwan; ^9^ Institute of Genomics and Bioinformatics National Chung Hsing University Taichung Taiwan; ^10^ Department of Applied Chemistry National Chi Nan University Puli Nantou Taiwan; ^11^ Institute of Biopharmaceutical Sciences National Yang‐Ming Chiao Tung University Taipei Taiwan; ^12^ Department of Pathology and Laboratory Medicine, Perelman School of Medicine University of Pennsylvania Philadelphia Pennsylvania USA; ^13^ School of Medicine Chung Shan Medical University Taichung Taiwan; ^14^ Department of Life Science Tunghai University Taichung Taiwan; ^15^ Program in Translational Medicine National Chung Hsing University Taichung Taiwan; ^16^ Rong Hsing Research Center for Translational Medicine National Chung Hsing University Taichung Taiwan

**Keywords:** AML, chemosensitivity, leukaemic stem cells, MPO, TRH

## Abstract

Leukaemia stem cells (LSCs) are major contributors to chemoresistance in acute myeloid leukaemia (AML). Identifying potential biomarkers within LSCs that can predict chemosensitivity in AML is key. This prospective study involved 20 consecutive de novo AML patients who underwent ‘7 + 3’ induction therapy. The patients were divided into CR (*n* = 15) and non‐CR (*n* = 5) groups. Using single‐cell RNA sequencing, we examined the cellular states of bone marrow mononuclear cells from AML patients at diagnosis and identified LSC among these cells. Our results showed that in non‐CR AML patients, a significant increase in the proportion of immature cells during haematopoiesis within the AML cell populations was observed. Moreover, the expression of myeloperoxidase (MPO) (log_2_ fold‐change = 0.89; adjusted *p* < 0.0001) and thyrotropin‐releasing hormone (TRH) (log_2_ fold‐change = 0.65; adjusted *p* < 0.0001) was higher within LSCs in the CR group than in the non‐CR group. Furthermore, patients with higher expression of MPO and TRH demonstrated improved relapse‐free survival (*p* = 0.002 for MPO; *p* = 0.009 for TRH) and overall survival (*p* = 0.002 for MPO; *p* < 0.001 for TRH). The connection between MPO or TRH and chemosensitivity could be linked with the downregulation of transforming growth factor and the upregulation of interferon‐α. In conclusion, MPO and TRH in LSCs could serve as chemosensitivity biomarkers in AML.

**Abbreviations**
AML: acute myeloid leukaemialeukemia
AUC: area under the curve
B: mature B cells
cDC: conventional dendritic cells
CR: complete remission
CTL: cytotoxic T lymphocytes
DEGs: differentially expressed genes
earlyEry: early erythroblasts
GMP: granulocyte‐macrophage progenitor cells
GSVA: gene set variation analysis
HSC: haematopoietichematopoietic stem cells
IHC: immunohistochemistry
lateEry: late erythroblasts
LSCs: leukaemialeukemia stem cells
Mono: monocytes
MPO: myeloperoxidase
NK: natural killer cells
OS: overall survival
OXPHOS: oxidative phosphorylation
pDC: plasmacytoid dendritic cells
Plasma: plasma cells
Prog: progenitor cells
ProMono: promonocytes
RFS: relapse‐free survival
scRNA‐seq: single‐cell RNA sequencing
T: naïve T cells
TRH: thyrotropin‐releasing hormone
UMI: unique molecular identifier

## 1 Background

Acute myeloid leukaemia (AML), with an annual age‐adjusted incidence rate of 4 per 100,000 people in the United States, is the most prevalent form of leukaemia in adults [1]. While radiation and chemotherapy exposure are associated with secondary AML, the precise pathophysiology of de novo AML remains unclear. A key mechanism underlying AML involves the abnormal clonal expansion of immature myeloid blasts resulting from the uncontrolled proliferation and immature differentiation of haematopoietic stem cells (HSC). In the case of chemotherapy‐eligible patients with AML, the primary treatment approach aims to achieve complete remission (CR) through induction chemotherapy, followed by consolidation chemotherapy to prevent potential disease relapse. Particularly in patients with specific genetic mutations or high‐risk cytogenetics, allogeneic haematopoietic stem cell transplantation has shown significant efficacy in reducing the risk of AML relapse and improving overall survival (OS) [2]. Nevertheless, attaining CR remains the initial and pivotal therapeutic goal for ensuring long‐term survival in individuals with AML.

The ‘7 + 3’ regimen, consisting of cytarabine 100 for 7 days and idarubicin 12 mg/m^2^ for 3 days, is the established standard induction therapy for newly diagnosed, chemotherapy‐eligible de novo AML, with a complete CR rate of 70% [2]. Patients with AML who fail to achieve CR following ‘7 + 3’ induction therapy experience a poor prognosis. Several clinical features, including high‐risk cytogenetics, advanced age, and leukocytosis, serve as readily identifiable factors for predicting the success of ‘7 + 3’ induction [3]. Furthermore, through a functional approach, our previous RNA‐seq data have revealed independent associations between the failure of ‘7 + 3’ induction in de novo AML and key pathways such as myc, mitochondrial oxidative phosphorylation (OXPHOS), mammalian target of rapamycin, and leukaemia stemness [4].

Among the various factors implicated in chemoresistance in de novo AML, leukaemia stem cells (LSCs) assume a significant role. Our previous study utilised a single‐cell RNA sequencing (scRNA‐seq) approach, revealing that AML cells exhibit either responsiveness or resistance to the ‘7 + 3’ induction regimen originating from a heterogeneous population of cancerous haematopoietic stem cells. Additionally, we observed that haematopoiesis arrest manifests at an earlier stage in chemoresistant AML cells [5]. The genetic biomarkers associated with LSCs and their role in predicting chemoresistance to the ‘7 + 3’ induction, as well as the underlying mechanisms of these genes, are currently unclear and require further investigation.

The present study utilised an in silico methodology to identify cells exhibiting elevated levels of stemness by employing three gene sets linked to LSCs. These cells, referred to as LSCs, were employed to discern genetic molecules capable of improving the chemosensitivity of de novo AML patients undergoing the ‘7 + 3’ induction. The discovered molecules were subsequently validated through the utilisation of external datasets. Furthermore, pathway, trajectory and cell–cell interaction analyses were conducted to investigate the potential mechanisms underlying these identified biomarkers. The data obtained from in silico analysis were further validated through in vitro experiments. Our findings can potentially guide induction strategies for newly diagnosed AML patients.

## 2 Methods

### 2.1 Patients

The study prospectively enrolled 20 patients with consecutive de novo AML, who underwent ‘7 + 3’ or ‘7 + 3’‐like induction therapy from March 2020 to June 2021. These patients were categorised into two groups based on their bone marrow status, assessed approximately 28 days post‐induction chemotherapy: the CR group (*n* = 15) and the non‐CR group (*n* = 5). Clinical features of the CR and non‐CR groups were compared, and the results are presented in Table S1. Notably, our previous study reported data on 13 out of these 20 patients [5].

### 2.2 Sample Preparation and Sequencing

Bone marrow samples were collected from these 20 patients at diagnosis. To collect AML cells, we employed a BD Vacutainer CPT mononuclear cell preparation tube (Franklin Lakes, NJ, USA) to enrich the concentration of myeloblast cells from the bone marrow sample. After performing density gradient centrifugation, the mononuclear cells were isolated and suspended in CELLBANKER (Zenogen Pharma, Fukushima, Japan). Subsequently, the cells were subjected to overnight freezing at −80°C and stored in liquid nitrogen for future use.

**FIGURE 1:**
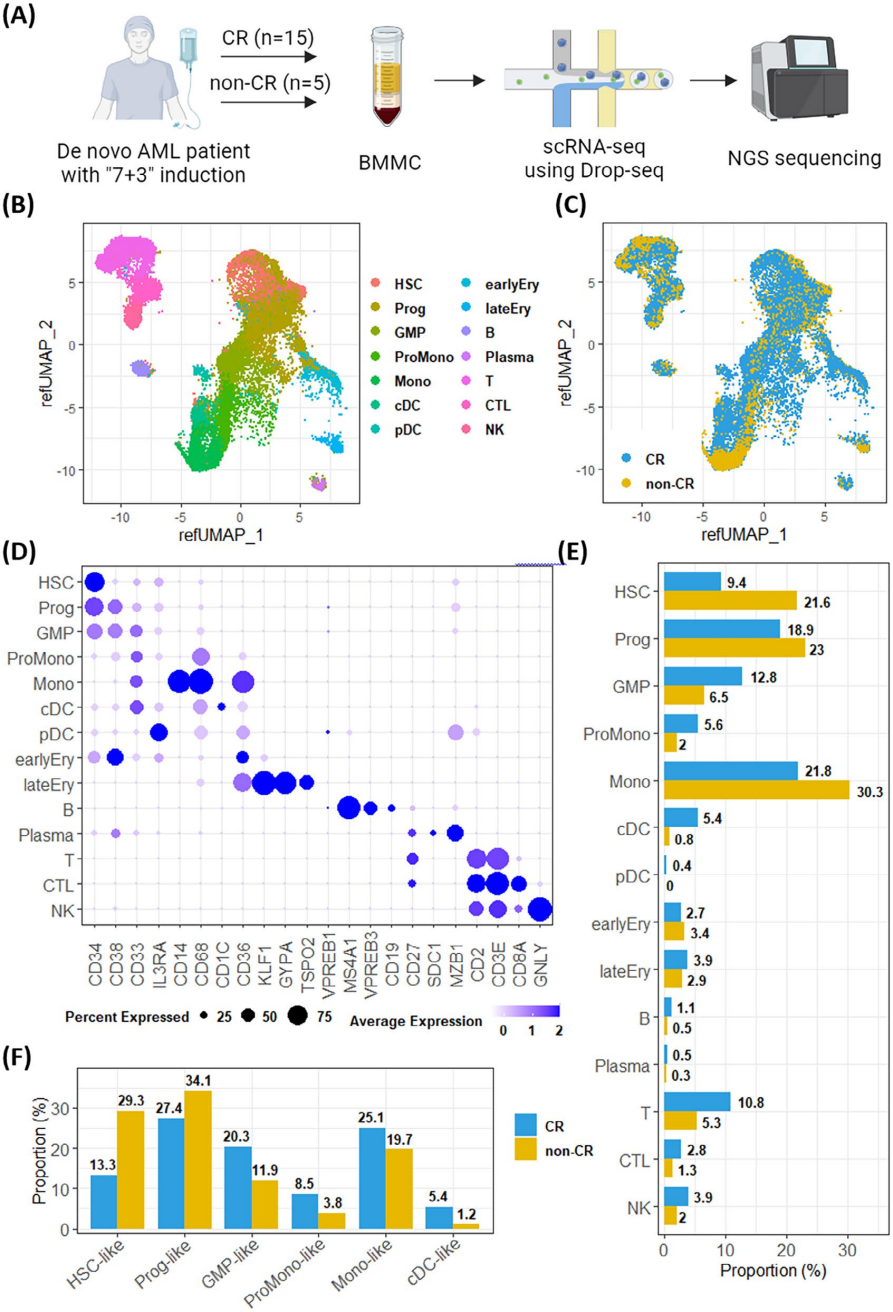
Mononuclear cells from acute myeloid leukaemia patients with complete remission (CR) or non‐CR. (A) Schematic overview of the patients and single‐cell RNA sequencing (scRNA‐seq) experiment. (B, C) We used GSE116256 as a reference to map our scRNA‐seq data to identify malignant cells and cell types. The distributions of (B) different types of cells and (C) cells from CR/ non‐CR patients were presented in Umap plots. (D) The dot plot presented the average expression and percent expression of marker genes in each cell type. (E, F) The bar charts showed the proportions of different cell types among (E) overall cells and (F) malignant cells separately in both CR patients and non‐CR patients.

The sample preparation was performed as previously described [5]. In brief, cryopreserved bone marrow samples were thawed in a 37°C water bath for 1 min, followed by treatment with Roswell Park Memorial Institute (RPMI) media supplemented with 10% fetal bovine serum and 10 ng/mL DNaseI to prevent cell aggregation. Subsequently, each bone marrow specimen's cell suspension was labelled with Cell Multiplex oligos (10× Genomics CG000391, Rev. B), and 4–7 samples were multiplexed onto a 10× Genomics Chromium Single Cell Instrument (10× Genomics, Pleasanton, CA, USA). Libraries were prepared using Chromium Next GEM Single Cell 3' Reagent Kits v3.1 (Dual Index, CG000388, Rev. A, Pleasanton, CA, USA) in accordance with the manufacturer's instructions. Finally, the libraries were sequenced on an Illumina NovaSeq 6000 platform to generate the fasta files (Figure 1A).

### 2.3 Single‐Cell Data Processing and Identifying Leukaemic Stem Cells

The scRNA‐seq data were processed and integrated using Cell Ranger Suite 6.0.1 along with the GRCh38 reference genome, generating unique molecular identifier (UMI) count matrices. Following barcode demultiplexing, the UMI matrices were imported into the R package Seurat (version 4.0.1) [6], which is an R toolkit utilised for quality control, analysis, and exploration of scRNA‐seq data. To ensure data quality, several criteria were employed. Cells expressing fewer than 500 genes, with total UMI counts below 1000 or over 10% of total UMI counts in the mitochondrial genome, were considered low quality or indicative of dead or dying cells. Doublets within each sample were identified using DoubletFinder [7]. These identified cells were subsequently excluded from further analyses. Normalisation was performed to mitigate cell‐to‐cell differences in transcript capture efficiency by scaling total UMI counts to a value of 10,000. Additionally, each expression value was log_2_‐transformed, adding one count to prevent undefined values resulting from zero counts.

We employed the Seurat pipeline to determine cell types in each sample by aligning the scRNA‐seq data with annotated scRNA‐seq data from healthy donors in the GSE116256 dataset, which served as a reference (Figure 1B) [8]. We performed dimensionality reduction using the top 50 principal components to establish anchors between our dataset and the GSE116256 dataset.

To identify malignant cells, we aligned various cell types from each sample with annotated malignant cells obtained from AML patients in the GSE116256 dataset. Subsequently, we employed Seurat's shared nearest neighbour algorithm to identify clusters of malignant cells and selected the cluster exhibiting the highest gene set variation analysis (GSVA) scores [9], which were calculated based on three gene sets associated with LSCs: LSC‐Ng [10], LSC‐R [11], and LSC52 [12], respectively. This study designated the cluster with the highest sum of the three GSVA scores as LSCs.

### 2.4 Trajectory Analysis

The trajectory analysis of malignant cells was performed using the R package Monocle [13]. Using Seurat's ‘FindAllMarkers’ function, we identified 889 genes specific to each cell type, satisfying the criteria of an adjusted *p*‐value < 0.05 and an average log_2_ fold change > 0.5. These genes were subsequently employed to arrange the cells in a specific order. To reduce dimensionality, we employed the DDRTree method. Pseudotime, representing the inferred developmental progression, was assigned to each cell, and scatter plots were generated to visualise the expression changes of genes along the cellular developmental trajectory.

### 2.5 Pathway Analysis

For each sample, GSVA was used to evaluate the enrichment level of a priori‐defined gene set [9], as implemented in the R package GSVA. Our analysis utilised the hallmark gene sets from the Molecular Signatures Database (MSigDB; https://www.gsea‐msigdb.org/gsea/msigdb/), along with three pre‐defined gene sets associated with LSCs [14]. We computed GSVA scores for each cell to estimate the enrichment levels of the gene sets obtained from MSigDB and the three LSC‐related gene sets. To identify differentially enriched gene sets between patients in CR and those in non‐CR, we conducted a *t*‐test using a Bonferroni‐corrected adjusted *p*‐value threshold of 0.05.

### 2.6 TCVGH, BeatAML2, and TCGA Datasets

We employed the TCVGH dataset [15] and acquired the BeatAML2 and TCGA datasets for validation cohorts [16, 17]. In the case of the TCVGH dataset, we aligned RNA‐seq reads to the GRCh38 reference from the Broad Institute (Homo_sapiens_assembly38_noALT_noHLA_noDecoy_ERCC.fasta) using STAR. Before alignment, we preprocessed the reads by removing sequences with adaptor remnants or a significant proportion of ambiguous bases [18]. RSEM was used to estimate gene expression levels based on the ERCC spike‐in reference annotation (gencode.v34.GRCh38.annotation.ERCC.gtf) [19]. The raw count data and clinical information for patients in the BeatAML2 dataset were retrieved from the official BeatAML2 website (https://biodev.github.io/BeatAML2/). Our analysis encompassed 255 de novo AML patients who had received standard chemotherapy. To distinguish between patients achieving CR or CR with incomplete blood count recovery and those who did not, we employed the edgeR method to identify differentially expressed genes [20]. In the case of the TCGA‐LAML dataset, we obtained the RNA‐seq data, which had been log_2_ (x + 1) transformed and RSEM normalised, along with clinical information for 173 patients. This data was sourced from UCSC Xena (https://tcga.xenahubs.net). Relapse‐free survival (RFS) was defined as the duration from sample collection to the first occurrence of relapse. The OS was determined based on the curated OS data available through Xena [21]. Cox regression analysis was used to assess the association between RESM normalised count and survival data.

### 2.7 In Vitro Validation

We utilised MOLM‐13 and HL‐60 AML cells for in vitro validation. These two cell lines were obtained from the American Type Culture Collection and maintained in a humidified incubator at 37°C with 5% CO_2_. The cells were cultured in Iscove's modified Dulbecco's medium supplemented with 10% fetal bovine serum, 100 units/mL penicillin, and 100 μg/mL streptomycin. Both cell lines were initially seeded at a density of 10^5^ cells/mL and cultured for 72 h to reach the steady‐state growth phase. We used Western blotting to validate the potential mechanisms underlying MPO and TRH‐related chemosensitivity in AML. Additionally, the trypan blue exclusion assay was employed to confirm the phenomenon that MPO and TRH increase AML chemosensitivity. Detailed protocols for these in vitro experiments are provided in the supplemental methods.

## 3 Results

### 3.1 Patients With AML Not Achieving CR Exhibited an Accumulation of Immature Cells During the Haematopoiesis

A total of 41,945 cells were analysed by the scRNA‐seq from 20 AML patients. The data with annotated reference data from healthy donors in the GSE116256 dataset. The cells were annotated into 14 major cell types, including HSC, progenitor cells (Prog), granulocyte‐macrophage progenitor cells (GMP), promonocytes (ProMono), monocytes (Mono), conventional dendritic cells (cDC), plasmacytoid dendritic cells (pDC), early erythroblasts (earlyEry), late erythroblasts (lateEry), mature B cells (B), plasma cells (Plasma), naïve T cells (T), cytotoxic T lymphocytes (CTL) and natural killer cells (NK) (Figure 1B). The distribution of CR and non‐CR cells is presented in Figure 1C. Canonical marker genes were used to confirm the cell types (Figure 1D) [5]. We observed higher percentages of HSC, Prog and Mono cell types in the non‐CR group than in the CR group (21.6% vs. 9.4% for HSC; 23% vs. 18.9% for Prog; 30.3% vs. 21.8% for Mono). Conversely, the GMP, ProMono and cDC proportions were lower in non‐CR patients than in CR patients (Figure 1E). However, these differences might be influenced by the presence of non‐malignant or normal cells. To further investigate the variations in the proportion of malignant cells between non‐CR and CR patients, we identified malignant cells by aligning HSC, Prog, GMP, ProMono, Mono, and cDC from each sample with annotated malignant cells obtained from AML patients in the GSE116256 dataset. We referred to the malignant cells in these categories as HSC‐like, Prog‐like, GMP‐like, ProMono‐like, Mono‐like, and cDC‐like. Our analysis revealed higher proportions of HSC‐like and Prog‐like populations in non‐CR patients (29.3% vs. 13.3% for HSC‐like; 34.1% vs. 27.4% for Prog‐like) than in CR patients, while the opposite was true for the other cell types (Figure 1F). These findings suggest that non‐CR AML patients show an accumulation of immature cells during early haematopoiesis within the AML cell populations.

### 3.2 Impact of LSC Identification and Cell Cycle on Chemoresistant AML Cells

**FIGURE 2:**
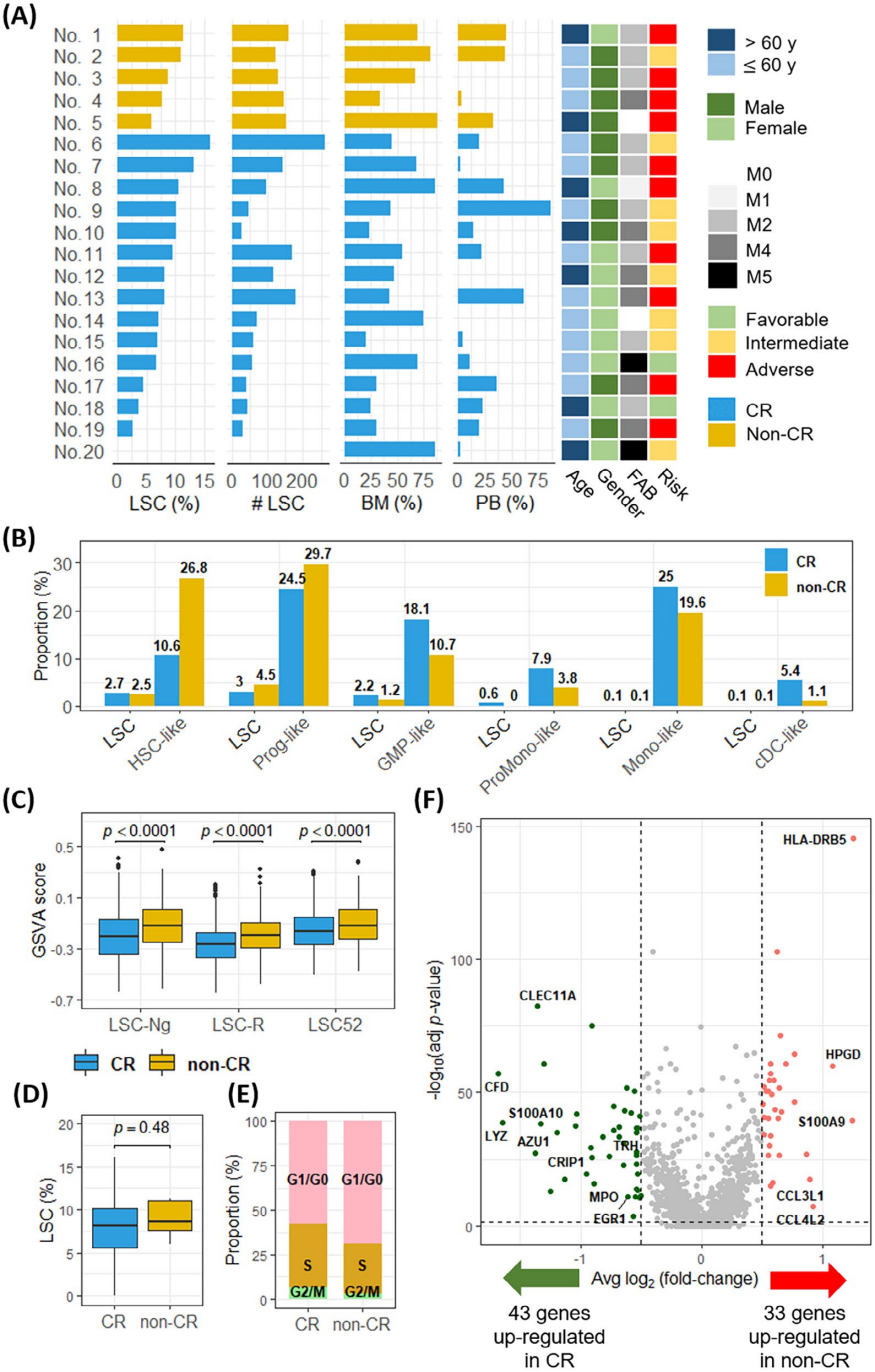
Landscape of leukaemic stem cells (LSCs) in patients with and without complete remission (CR). (A) The bar charts present the proportion and number of LSCs in each acute myeloid leukaemia patient, and the heatmap shows their clinical information. (B) The bar charts present the proportion of LSCs identified from malignant cells in each cell type. (C) The boxplot shows that non‐CR patients exhibited higher levels of stemness than CR patients, based on the gene set variation analysis score estimated from three LSC‐related gene sets. (D) The proportions of LSCs among malignant cells were compared between CR and non‐CR patients. (E) The proportions of LSCs across different cell cycle phases are presented in both CR and non‐CR patients. (F) The volcano plot presents the differential expression of 76 genes between LSCs from non‐CR and CR patients. The vertical dotted line represents the adjusted *p*‐value threshold of 0.05, and the horizontal dotted line represents the average log_2_ fold change threshold of 0.5.

Given the accepted role of LSCs in contributing to chemoresistance [22, 23, 24], we proceeded to identify LSC clusters in silico from malignant cells using Seurat and evaluated three gene sets associated with LSCs (Figure S1). Cells exhibiting the highest GSVA scores within each patient were designated as LSCs. Although the proportions of LSCs varied among patients, 0%–16% (Figure 2A), the proportions derived from malignant cells remained relatively low (Figure 2B). Notably, we observed that non‐CR patients displayed higher GSVA scores than did CR patients (*p* < 0.0001 for LSC‐Ng, LSC‐R, and LSC52) (Figure 2C). However, no significant difference in the proportion of LSCs was found between these two groups (*p* = 0.48) (Figure 2D). To investigate the relationship between cell cycle phases and chemoresistance, we assessed the proportions of cell cycle phases in LSCs using canonical markers in Seurat. Our findings indicated that non‐CR patients had a higher proportion of LSCs in the G0/G1 phase (69% vs. 58%) and a lower combined proportion of cells in S phase and G2/M phase (31% vs. 42%) than did CR patients (Figure 2E). These findings imply that in non‐CR patients, LSCs tend to become arrested in the G0/G1 phase, potentially resulting in decreased cellular division, which is a known factor linked to chemoresistance [25].

### 3.3 Biomarkers to Predict Chemosensitivity in AML

**FIGURE 3:**
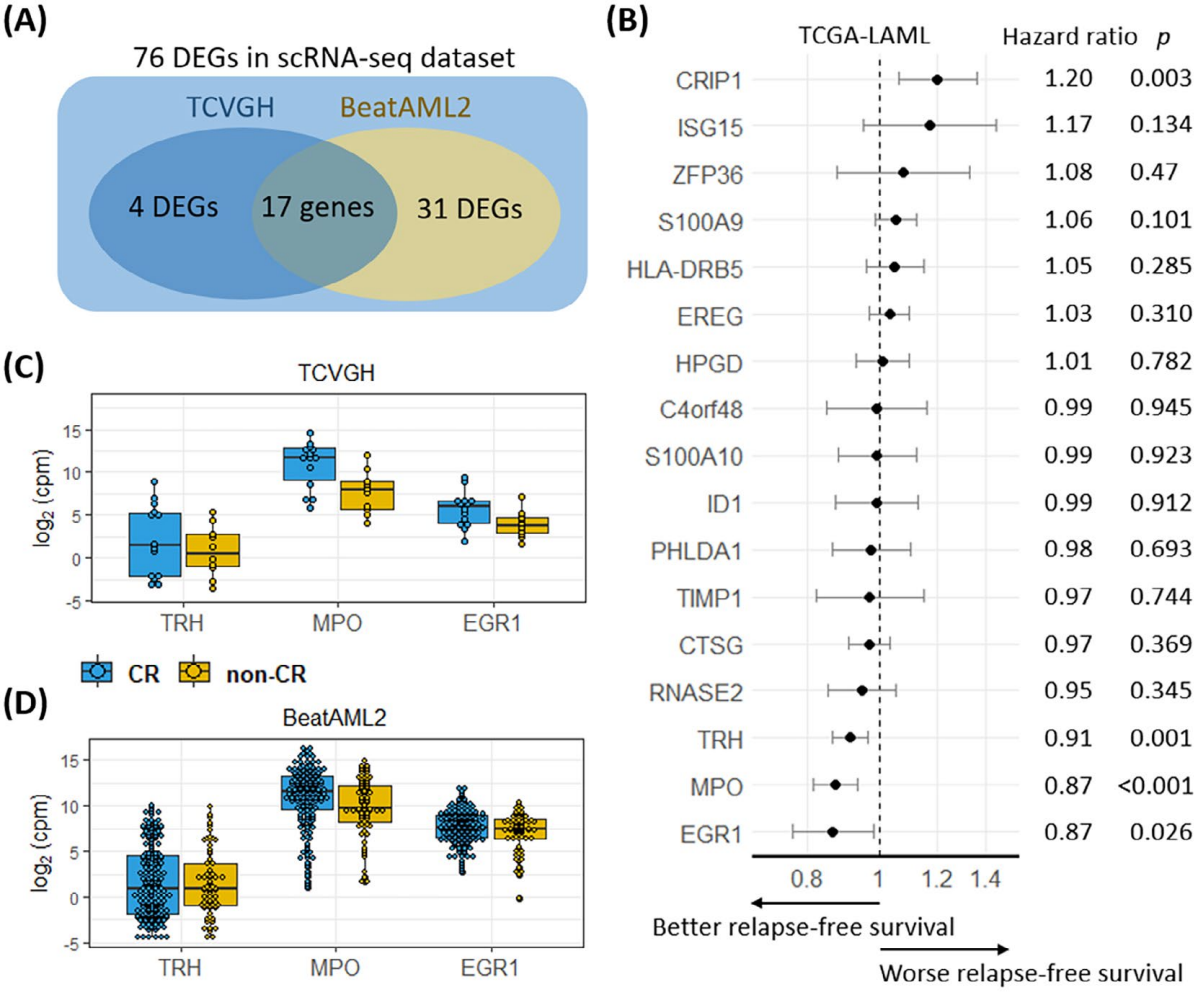
Validation in TCVGH, BeatAML2, and TCGA‐LAML cohorts. (A) The Venn diagram shows the overlapping differentially expressed genes (DEGs) between the BeatAML2 and TCVGH cohorts based on the DEGs from the scRNA‐seq dataset. A total of 17 genes were identified to be significantly differentially expressed in the scRNAseq dataset, BeatAML2, and TCVGH cohorts simultaneously. (B) The forest plot shows the hazard ratios (HRs) and corresponding *p*‐values from the univariate Cox regression analysis for relapse‐free survival (RFS) in the TCGA‐LAML cohort. Three genes (MPO, TRH, and EGR1) were identified to be significantly associated with RFS. (C, D) The boxplots present the expression level of these four genes, which were differentially expressed between CR and non‐CR patients, in (C) the TCVGH and (D) BeatAML2 cohorts.

**FIGURE 4:**
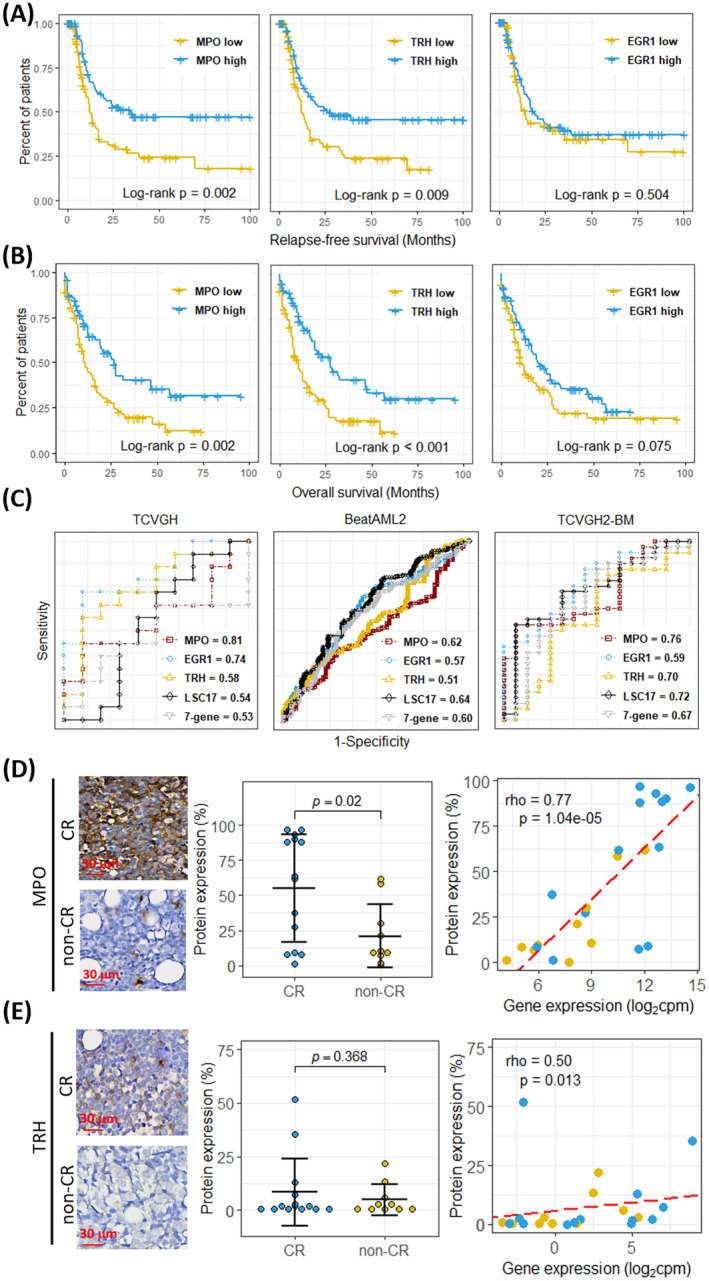
Performance of predicting chemoresistance in TCVGH, BeatAML2, and an external dataset. (A, B) Kaplan–Meier plots show that the CR‐specific genes TRH, MPO, and EGR1 were protective factors for (A) relapse‐free survival and (B) overall survival. The high and low expression groups were determined based on the median expression levels. (C) The predictive performance of TRH, MPO, EGR1, LSC17 score, and 7‐gene score in predicting chemoresistance was assessed using receiver operating characteristic curves in the TCVGH, BeatAML2, and TCVGH2‐BM datasets. The area under the curve values are indicated in the lower‐right corner of each plot. (D) and (E) Immunohistochemical (IHC) staining of candidate protein expression in AML patient specimens. Representative images of IHC staining for MPO (D) and TRH (E) at ×400 magnification, displaying sections from patients with complete remission (CR) and non‐complete remission (non‐CR). The bar graph depicts the percentage of staining in the CR and non‐CR groups, with statistical significance determined by a *t*‐test. The scatter plot illustrates the correlation between protein expression (%) and gene expression (log₂ cpm), with the Spearman's correlation coefficient (Rho) and *p*‐value (*p*) indicated.

To identify potential biomarkers associated with chemosensitivity in AML patients undergoing ‘7 + 3’ induction, we conducted a differentially expressed genes (DEGs) analysis among LSCs between the non‐CR and CR groups, revealing 76 differentially expressed genes (*p*‐value < 0.01, Log_2_ fold change > 0.5) (Figure 2F and Table S2). To validate these findings, we performed further analysis using independent datasets of TCVGH [15], BeatAML2 [16], and TCGA‐LAML [17] as the validation cohorts. Among these 76 DEGs, we observed that 17 genes exhibited statistically significant differential expression in the TCVGH and BeatAML2 datasets (Figure 3A). Subsequently, by employing Cox regression analysis on the TCGA‐LAML dataset, we identified 17 genes that exhibited associations with risks of RFS. Importantly, MPO, TRH, and EGR1 exhibited hazard ratios < 1, demonstrating a substantial association with extended RFS (Figure 3B). These data were externally validated by the TCVGH (Figure 3C) and BeatAML2 (Figure 3D) datasets. Additionally, after categorising patients into high and low expression groups based on the median expression levels of MPO and TRH in the TCGA‐LAML dataset, we observed that patients with higher levels of MPO and TRH expression exhibited significantly improved RFS (*p* = 0.002 for MPO; *p* = 0.009 for TRH) (Figure 4A) and OS (*p* = 0.002 for MPO; *p* < 0.001 for TRH) (Figure 4B). However, EGR1 did not show a statistically significant difference in survival. These findings suggest that MPO and TRH, but not EGR1, have the potential to serve as biomarkers within LSCs associated with improved survival in AML.

Further, to assess the predictive performance of MPO and TRH in response to ‘7 + 3’ induction therapy compared to the LSC17 and 7‐gene scores, the area under the curve (AUC) was used to evaluate the predictive efficacy. We evaluated the LSC17 score and 7‐gene score in the TCVGH and BeatAML2 datasets [4, 10], as well as an external dataset of TCVGH2‐BM [4], comprising bone marrow samples from patients at TCVGH other than the current study cohort. In the TCVGH dataset, the highest AUC was observed for MPO (AUC = 0.81), followed by EGR1, TRH, LSC17 score, and 7‐gene score. In the BeatAML2 dataset, however, the highest AUC was found for LSC17 (AUC = 0.64), followed by MPO (AUC = 0.62) (Figure 4C). Notably, in the TCVGH2‐BM database that sampled mononuclear cells isolated from the bone marrow of patients before chemotherapy, MPO with AUC = 0.76 was the most discriminative predictor. Combining the analysis data of these three databases, the MPO gene is poised to be an effective biomarker for predicting treatment response in AML patients undergoing ‘7 + 3’ induction therapy.

To correlate MPO and TRH expression with AML immunophenotype at diagnosis, immunohistochemistry (IHC) was used to assess the protein expression levels of MPO and TRH in patients' bone marrow samples. The ImageXpress PICO (Molecular Devices, San Jose, CA, USA) analysis revealed that the proportion of MPO‐stained cells in the CR and non‐CR groups was 54.90% ± 10.23% and 20.92% ± 7.12%, respectively (*p* = 0.0125) (Figure 4D). Additionally, correlation analysis between MPO gene expression from bulk RNA sequencing and protein expression from IHC revealed a strong positive correlation (Spearman's correlation coefficient; Rho = 0.77; *p* = 1.04e‐05) (Figure 4D). In contrast, the IHC staining proportions of TRH were 8.41% ± 4.19% in the CR group and 4.93% ± 2.28% in the non‐CR group, suggesting a trend toward higher expression in the CR group; however, this difference was not statistically significant (*p* = 0.4734) (Figure 4E). Additionally, correlation analysis between TRH gene expression and IHC protein expression demonstrated a significant association (Spearman's correlation coefficient; Rho = 0.50; *p* = 0.0133) (Figure 4E).

### 3.4 Annotation of MPO and TRH as Related to CR Through Pathway and Trajectory Analysis

**FIGURE 5:**
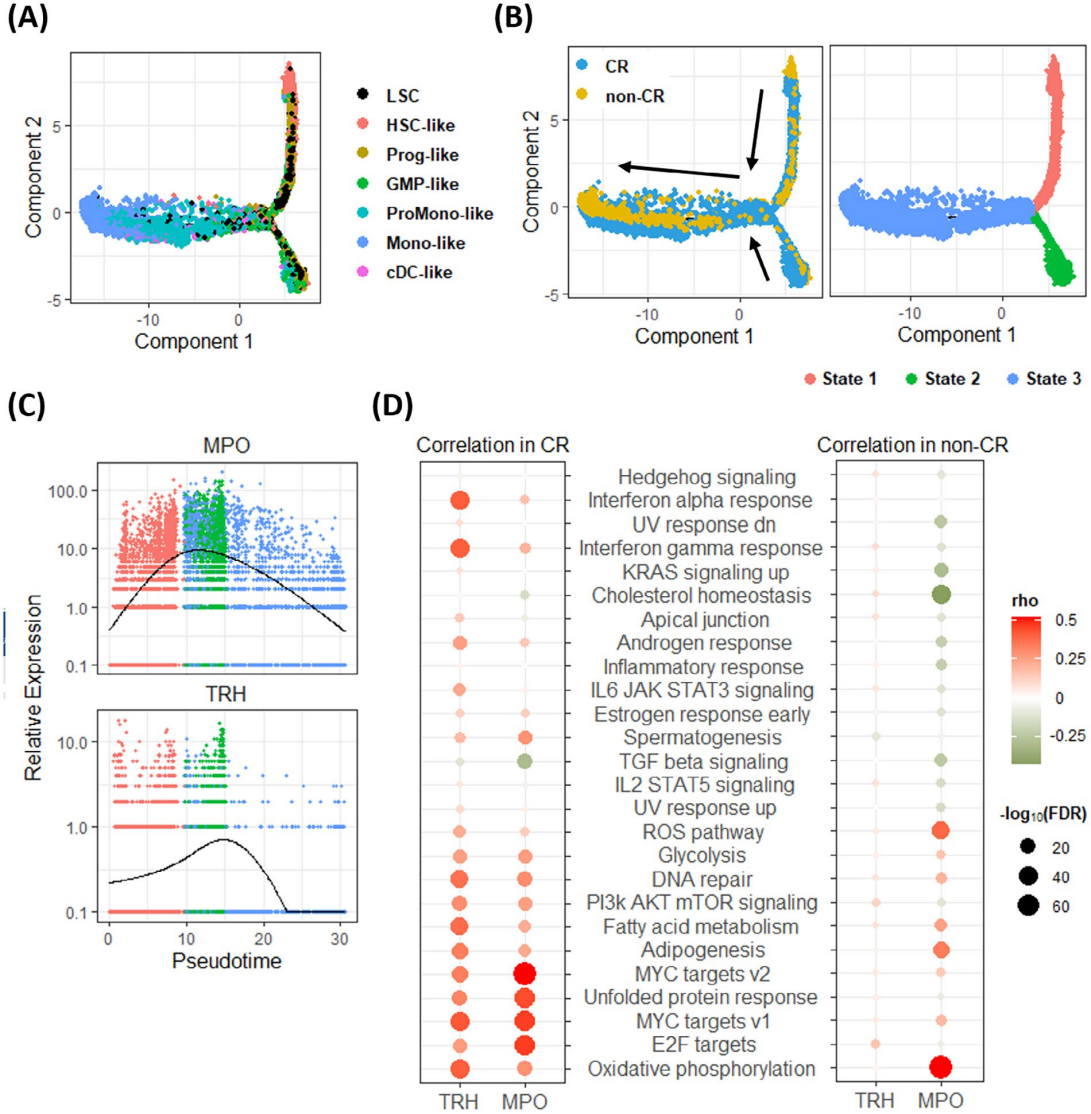
Annotation of CR‐specific genes using trajectory and pathway analysis. (A) The monocle‐generated plot presents the differentiation trajectory of malignant cells within the context of haematopoiesis. (B) Utilising pseudotime analysis to assess malignant cells, we categorised them into three distinct states. State 1 exhibited an enrichment of malignant cells primarily from non‐CR patients, whereas state 2 displayed an enrichment of malignant cells predominantly from CR patients. (C) Scatterplots present the relative expression levels of TRH and MPO for the different states arranged along pseudotime. The colour indicating the different states is shown in (B). (D) The dot plots illustrate Spearman correlations between gene expression specific to CR and pathway enrichment scores computed via GSVA. Positive correlations are depicted in red, while negative correlations are denoted in green. The size of the circles corresponds to the −log_10_ false discovery rate.

We employed the monocle algorithm to construct single‐cell trajectories to investigate the differentiation trajectory of malignant cells within the context of haematopoiesis. Our analysis revealed a significantly higher abundance of HSC‐like and Prog‐like cells in the top root than in the bottom root (Figure 5A). Of note, the upper root, designated as State 1, displayed a higher proportion of malignant cells from non‐CR patients, while the lower root, designated as State 2, exhibited an enrichment of malignant cells from CR patients (Figure 5B and Figure S2). These results not only corroborated our previous findings concerning the elevated presence of HSC‐like and Prog‐like cells in non‐CR patients but also suggested that chemoresistant AML cells may undergo similar haematopoietic processes to those of chemosensitive AML cells.

Additionally, we investigated the potential role of MPO and TRH within LSCs in enhancing the chemosensitivity of ‘7 + 3’ induction therapy. The expression levels of MPO and TRH initially increased before reaching State 2, followed by a subsequent decrease as pseudotime advanced. Notably, the expression changes of MPO over pseudotime exhibited similarities to those of TRH (Figure 5C). These results underscore the potential significance of MPO and TRH as predictive biomarkers for assessing the response to ‘7 + 3’ induction therapy. To assess the expression levels of MPO and TRH in various cell types within AML, our data indicated that the gene expression levels of MPO and TRH, both in the overall LSC population and in the early haematopoietic stages of HSC‐like, prog‐like, and GMP‐like cells, were higher in CR patients than in non‐CR patients. However, in the later stages of haematopoiesis, their expression was predominantly observed in non‐CR patients (Figure S3).

To delve deeper into the functional characterisation of LSCs, we performed GSVA using hallmark gene sets sourced from the MSigDB to analyse the correlation between AML‐related regulatory pathways and CR‐specific genes. When contrasting LSCs in non‐CR patients with those in CR patients, we observed a pronounced enrichment of the Hedgehog signalling pathway in non‐CR patients (Figure S4). Conversely, CR patients exhibited a significant enrichment of the OXPHOS pathway and a higher fraction of LSCs in the S and G2/M phases than non‐CR patients (Figure 2D). Moreover, the TRH gene exhibited specific expression in regulatory pathways associated with CR patients and was nearly absent in the non‐CR group (Figure 5D). To be precise, LSCs expressing TRH from CR patients displayed a significant enrichment in interferon alpha and gamma responses (Figure 5D, left panel). Furthermore, we noted a substantial enrichment of MYC targets v1 and v2 as well as the E2F target pathway, which negatively regulated the transforming growth factor (TGF)‐β pathway in LSCs expressing MPO from CR patients (Figure 5D, left panel). Conversely, LSCs expressing MPO from non‐CR patients exhibited a noteworthy enrichment in cholesterol homeostasis, with a negative correlation (Figure 5D, right panel).

### 3.5 In Vitro Validation

**FIGURE 6:**
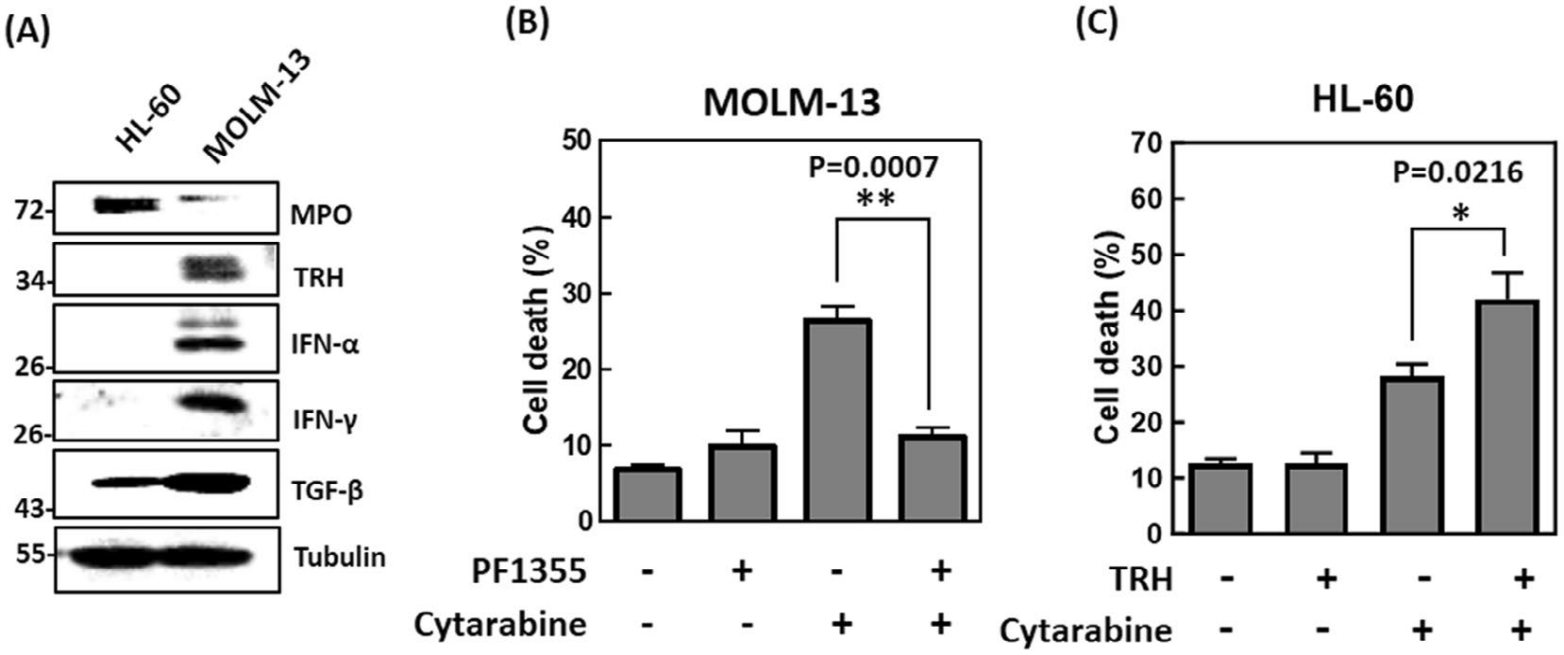
In vitro validation. (A) Western blot analysis of MPO, TRH, IFN‐α, IFN‐γ, TGF‐β, and tubulin (internal control) protein expression in HL‐60 and MOLM‐13 cells. (B) Percentage of cell death in MOLM‐13 cells after 24 h of treatment with 2 μM PF1355 (MPO inhibitor) and/or 1 μM cytarabine. (C) Percentage of cell death in HL‐60 cells after 24 h of treatment with 100 nM TRH and/or 1 μM cytarabine.

Figure 6A illustrates the endogenous expression of MPO and TRH in MOLM‐13 and HL‐60 AML cells. Briefly, HL‐60 cells exhibited higher endogenous MPO expression compared to MOLM‐13 cells, while MOLM‐13 cells had higher endogenous TRH expression than HL‐60 cells. Notably, TGF‐β expression was also higher in MOLM‐13 cells than in HL‐60 cells. Additionally, MOLM‐13 cells with higher TRH expression exhibited greater levels of IFN‐α and IFN‐γ compared to HL‐60 cells. These findings partially support the trajectory pathway analyses shown in Figure 5.

We further validated the phenomenon that MPO and TRH increased the chemosensitivity in AML. As shown in Figure 6B, MOLM‐13 cells were treated with the MPO inhibitor PF1355 and cytarabine. Treatment with cytarabine alone increased cell death from 6.86% ± 0.62% to 26.49% ± 1.83% (*p* = 0.0001). However, co‐treatment with PF1355 and cytarabine significantly reversed the percentage of cell death from 26.49% ± 1.83% to 11.09% ± 1.39% (*p* = 0.0007), indicating that MPO inhibition reduces cell sensitivity to chemotherapy. In contrast, in HL‐60 cells, treatment with TRH alone did not affect cell death. However, co‐treatment with TRH and cytarabine for 24 h resulted in a significant increase in cell death, from 28.18% ± 2.26% with cytarabine alone to 41.95% ± 4.73% with the combination (*p* = 0.0216) (Figure 6C). These results suggest that the presence of TRH substantially enhances cell sensitivity to chemotherapy.

## 4 Discussion

In the current study, we utilised scRNA‐seq to perform in‐depth cellular profiling and identified specific LSC subpopulations associated with chemoresistance. Our results demonstrated that non‐CR AML patients exhibited a notable increase in the proportion of immature cells during haematopoiesis within the AML cell populations. Furthermore, we proposed that MPO and TRH expression levels within LSCs could serve as reliable biomarkers for predicting the therapeutic response in AML patients undergoing the ‘7 + 3’ induction therapy.

Our data revealed that MPO expression was significantly higher in CR patients than in non‐CR patients. A study by Hosseini et al. [26] suggested that MPO plays a role in diminishing resistance to cytarabine in AML patients by perturbing mitochondrial redox balance. These findings align with our data, substantiating the significant association between elevated MPO expression within LSCs from AML patients and an increased likelihood of achieving CR. External validation further solidified the substantial correlation between elevated MPO expression in AML patients and enhanced RFS (Figure 4A) as well as extended OS time (Figure 4B). Compared to LSC‐17 and the 7‐gene scores, MPO exhibited a higher AUC score in two patient datasets from TCVGH (Figure 4C). In the BeatAML2 validation, MPO achieved an AUC score of 0.62, which was slightly lower than that of LSC‐17 (AUC = 0.64). This difference may be attributed to variations in patient demographics across different countries. Nevertheless, these findings underscore the robust predictive potential of MPO for assessing chemosensitivity in AML.

In our mechanistic analysis, we demonstrated that MPO exerts a negative regulatory effect on the expression of the TGF‐β pathway in the CR group (Figure 5D), suggesting that MPO may function to inhibit the TGF‐β regulatory pathway. Accordingly, a previous study by Zhang et al. [27] established that TGF‐β activation is associated with heightened chemoresistance. Consequently, the suppression of the TGF‐β pathway by MPO may enhance the chemosensitivity of ‘7 + 3’ induction therapy in AML. While the in vitro experiments conducted in this study provided partial support for this hypothesis, further research is needed to fully validate it. Notably, LSCs from non‐CR patients exhibited significantly heightened Hedgehog signalling activity. Although the role of Hedgehog signalling in the development, maintenance, and expansion of LSCs has been previously documented in vitro and in vivo [28, 29], our study provides clinical evidence supporting the relevance of this phenomenon.

Alongside MPO, TRH may also hold the potential to serve as a predictive marker for outcomes in AML patients. In our present investigation, we observed a substantial elevation in TRH expression within LSCs from AML patients who attained CR. Gao et al. [30] previously established that TRH expression in AML has the potential to enhance the ELN 2017 risk stratification system and evaluate the survival of AML patients, which aligns with our findings. Moreover, the underlying pathway analysis unveiled a robust positive correlation between TRH and the interferon (IFN)‐α and IFN‐γ pathways. This observation aligns with prior research demonstrating a connection between the neuroendocrine and immune systems. Specifically, it has been shown that TRH stimulation results in significant activation of IFN‐γ, both in vivo and in vitro [31]. Furthermore, the independent prognostic value of IFN‐α activation via the TLR3‐TBK1‐IRF3 pathway has been established in AML [32]. Given that our results demonstrated a significant enhancement in RFS and OS rates in AML associated with elevated TRH expression levels (Figure 4A,B), we hypothesize that augmenting TRH expression within LSCs via the activation of the IFN‐α and IFN‐γ pathways is linked to increased chemosensitivity in AML, ultimately resulting in improved RFS and OS.

The primary limitation of our study stems from the relatively small number of patients included. Additionally, our investigation utilised an in silico methodology to explore potential pathways related to the roles of MPO and TRH in AML chemosensitivity, allowing us to identify associations but not to elucidate precise mechanisms. While the in vitro experiments in this study only provided partial support for the potential mechanisms of MPO and TRH in AML chemosensitivity, further in vitro and in vivo studies are essential to explore the detailed mechanistic interactions between MPO or TRH in LSCs and their impact on chemosensitivity in AML.

## 5 Conclusions

In conclusion, our findings revealed that non‐CR AML patients exhibited a significant increase in the proportion of immature cells during haematopoiesis within the AML cell populations. Moreover, the activation of numerous regulatory pathways within AML LSCs, including dysregulation of the OXPHOS pathway, perturbed cholesterol homeostasis, and aberrant MYC activity, has been associated with chemoresistance, thereby presenting formidable challenges in AML treatment. Additionally, we have proposed a hypothesis suggesting that MPO and TRH expression levels within LSCs could serve as robust biomarkers for predicting therapeutic responses in AML patients undergoing ‘7 + 3’ induction therapy. These two candidate factors also hint at the potential for pathway modulation, with negative TGF‐β regulation and positive IFN‐α regulation, which may ultimately enhance chemotherapy sensitivity and guide patients toward achieving CR.

## Author Contributions

**Chung‐Hsing Chen:** conceptualization (equal), formal analysis (equal), investigation (equal), writing – review and editing (equal). **Tsung‐Chih Chen:** investigation (equal), methodology (equal). **Ting‐Shuan Wu:** conceptualization (equal), investigation (equal), methodology (equal). **Tzu‐Hung Hsiao:** investigation (equal), methodology (equal), writing – review and editing (equal). **Jo‐Mei Maureen Chen:** methodology (equal). **Chi‐Ying F. Huang:** conceptualization (equal), writing – review and editing (equal). **Po‐Liang Cheng:** investigation (equal), methodology (equal), software (equal). **Jia‐Rung Tsai:** investigation (equal), methodology (equal). **Chieh‐Lin Jerry Teng:** conceptualization (equal), supervision (equal), writing – review and editing (equal).

## Ethics Statement

The Institutional Review Board of Taichung Veterans General Hospital approved this study in accordance with the current version of the Declaration of Helsinki (CF17319B).

## Consent

All participants provided informed consent before participating in the study. The patient details were de‐identified.

## Conflicts of Interest

Chieh‐Lin Jerry Teng received honorarium and consulting fees from Novartis, Roche, Pfizer, Takeda, Johnson and Johnson, Amgen, BMS Celgene, Kirin, and MSD. The other authors declare no conflicts of interest.

## Supporting information


Tables S1–S2.

**Figures S1–S4.**Characterisation of LSCs in a cohort of three patients who achieved CR and three patients who did not attain CR. The top set of samples represents those from patients with CR, while the bottom set pertains to patients without CR. In each sample, the UMAP plot showcases clusters identified through the SNN algorithm, while the boxplot highlights the cluster with the highest stemness levels, determined by calculating GSVA scores from three LSC‐related gene sets: LSC‐Ng, LSC‐R, and LSC52. The clusters within each boxplot are arranged based on the cumulative scores obtained from these assessments. Figure S2 Monocle‐generated plot depicting the differentiation trajectory of malignant cells. The pseudotime was estimated using Monocle, with cells depicted in a lighter colour to indicate a longer pseudotime. Figure S3 the mRNA expression of (A) MPO and (B) TRH between non‐CR and CR patients in malignant cells in each cell type. Figure S4 Comparison of annotated LSCs between patients who did not achieve CR and those who achieved CR. Bar charts illustrate the differential enrichment of hallmark gene sets within LSCs among non‐CR and CR patients. For each gene set, the *t*‐statistic derived from GSVA scores is reported, and multiple comparisons are adjusted using the Bonferroni correction. Hallmark gene sets with a significance level of 0.05 (Bonferroni‐corrected *p*‐value < 0.05) are highlighted. The gene sets are organised based on their respective *t*‐statistics.

## Data Availability

The scRNA‐seq data generated using Cell Ranger have been made publicly available in the GEO database (GSE240618). The processed data is available from Single Cell Portal (https://singlecell.broadinstitute.org/single_cell) (SCP2316).
