# Supplementary material for: Myeloperoxidase and Thyrotropin‐Releasing Hormone Within Leukaemia Stem Cells Increased Chemosensitivity in Acute Myeloid Leukaemia
Source: J Cell Mol Med. 2024 Dec 25;28(24):e70306. doi: 10.1111/jcmm.70306 (PMC11669111; doi:10.1111/jcmm.70306)
Supplement: Supplementary file 1 — Tables S1–S2. [file JCMM-28-e70306-s001.docx]

| **Supplementary Table S1. Clinical characteristics of 20 patients with acute myeloid leukeia** | | | | | | | | |
| --- | --- | --- | --- | --- | --- | --- | --- | --- |
| **Patient No.** | **Age (years)** | **Gender** | **Leukocyte (10^9^/L)** | **Blast in BM (%)** | **Blasts in PB (%)** | **FAB** | **Cytogenetics risk** | **CR or non-CR** |
| 1 | 62 | Female | 8.1 | 70 | 45 | M2 | Adverse | Non-CR |
| 2 | 35 | Male | 184.1 | 82 | 44 | M2 | Intermediate | Non-CR |
| 3 | 48 | Male | 24.9 | 68 | 0 | M2 | Adverse | Non-CR |
| 4 | 33 | Male | 33.7 | 34 | 3 | M4 | Adverse | Non-CR |
| 5 | 68 | Male | 5.2 | 89 | 33 | M0 | Adverse | Non-CR |
| 6 | 53 | Male | 4.7 | 45 | 20 | M2 | Intermediate | CR |
| 7 | 31 | Male | 1.1 | 69 | 2 | M2 | Adverse | CR |
| 8 | 60 | Female | 183.7 | 87 | 43 | M1 | Adverse | CR |
| 9 | 45 | Male | 45.3 | 44 | 87 | M2 | Intermediate | CR |
| 10 | 60 | Male | 5.3 | 24 | 14 | M4 | Intermediate | CR |
| 11 | 52 | Female | 35.1 | 55 | 22 | M2 | Adverse | CR |
| 12 | 64 | Female | 8.0 | 48 | 0 | M4 | Intermediate | CR |
| 13 | 51 | Female | 292.0 | 43 | 62 | M4 | Adverse | CR |
| 14 | 28 | Female | 1.3 | 75 | 0 | M0 | Intermediate | CR |
| 15 | 58 | Female | 161.3 | 20 | 5 | M2 | Intermediate | CR |
| 16 | 53 | Female | 70.1 | 70 | 11 | M5 | Favorable | CR |
| 17 | 42 | Male | 79.4 | 31 | 36 | M4 | Adverse | CR |
| 18 | 63 | Female | 12.5 | 25 | 23 | M2 | Favorable | CR |
| 19 | 41 | Male | 93.5 | 31 | 20 | M4 | Adverse | CR |
| 20 | 62 | Female | 60.8 | 87 | 2 | M5 | Intermediate | CR |
| BM: bone marrow; PB: peripheral blood; FAB: French-America-British; CR: complete remission | | | | | | | | |

| **Supplementary Table S2. The genes within LSCs with differential expression between CR and non-CR patients** | | | | |
| --- | --- | --- | --- | --- |
| **Symbol** | **avg_log2FC*** | **pct.1^†^** | **pct.2^‡^** | **adjusted *p*-value** |
| CLEC11A | -1.359 | 0.401 | 0.742 | 4.58E-83 |
| TYROBP | -0.910 | 0.847 | 0.934 | 7.57E-76 |
| XIST | -1.304 | 0.218 | 0.545 | 2.39E-61 |
| CFD | -1.685 | 0.435 | 0.680 | 7.92E-58 |
| H3F3B | -0.622 | 1.000 | 1.000 | 1.29E-52 |
| C4orf48 | -0.557 | 0.336 | 0.589 | 2.39E-51 |
| PHLDA1 | -0.725 | 0.093 | 0.340 | 1.85E-45 |
| TIMP1 | -0.640 | 0.789 | 0.841 | 5.20E-44 |
| ISG15 | -0.581 | 0.325 | 0.482 | 2.93E-43 |
| CSTA | -1.037 | 0.115 | 0.343 | 1.26E-42 |
| PVT1 | -0.514 | 0.135 | 0.263 | 4.87E-42 |
| LYZ | -1.652 | 0.567 | 0.719 | 1.90E-39 |
| S100A10 | -1.331 | 0.596 | 0.674 | 5.17E-39 |
| SRGN | -1.043 | 0.928 | 0.952 | 4.28E-38 |
| CAST | -0.687 | 0.368 | 0.547 | 5.66E-38 |
| POU4F1 | -0.542 | 0.021 | 0.221 | 2.40E-37 |
| HACD1 | -0.530 | 0.301 | 0.356 | 3.06E-37 |
| HIST1H4C | -0.728 | 0.903 | 0.948 | 1.44E-36 |
| IGFBP7 | -0.536 | 0.828 | 0.887 | 9.59E-36 |
| CTSG | -1.194 | 0.125 | 0.293 | 1.36E-35 |
| DUSP2 | -0.682 | 0.181 | 0.355 | 2.71E-34 |
| CST3 | -0.821 | 0.582 | 0.807 | 4.16E-34 |
| TRH | -0.646 | 0.153 | 0.392 | 8.04E-32 |
| PCDH9 | -0.915 | 0.001 | 0.137 | 7.32E-30 |
| ZEB2 | -0.538 | 0.769 | 0.844 | 2.11E-28 |
| AZU1 | -1.380 | 0.269 | 0.447 | 5.70E-28 |
| TSPO | -0.539 | 0.624 | 0.703 | 2.37E-27 |
| RNASE2 | -0.768 | 0.103 | 0.277 | 8.02E-27 |
| EREG | -0.911 | 0.110 | 0.312 | 2.35E-26 |
| APOC2 | -0.539 | 0.108 | 0.289 | 6.10E-24 |
| HIST1H1C | -0.645 | 0.671 | 0.794 | 1.16E-23 |
| CRIP1 | -0.958 | 0.720 | 0.749 | 2.25E-20 |
| TRGC2 | -0.534 | 0.184 | 0.386 | 4.46E-20 |
| ELANE | -1.136 | 0.093 | 0.243 | 5.67E-18 |
| MPO | -0.890 | 0.588 | 0.571 | 1.33E-16 |
| ZFP36 | -0.542 | 0.858 | 0.856 | 2.29E-14 |
| IER2 | -0.531 | 0.885 | 0.938 | 2.96E-14 |
| PRTN3 | -1.255 | 0.062 | 0.108 | 8.81E-14 |
| CXCR4 | -0.500 | 0.532 | 0.625 | 6.18E-12 |
| NFKBIA | -0.549 | 0.958 | 0.947 | 1.79E-11 |
| EGR1 | -0.610 | 0.288 | 0.436 | 1.83E-11 |
| JUNB | -0.512 | 0.880 | 0.927 | 4.39E-11 |
| ID1 | -0.564 | 0.290 | 0.366 | 4.17E-04 |
| HLA-DRB5 | 1.252 | 0.571 | 0.117 | 2.73E-146 |
| MT-ATP6 | 0.626 | 1.000 | 0.997 | 9.68E-104 |
| LIMS1 | 0.648 | 0.850 | 0.711 | 3.67E-72 |
| CLEC2B | 0.764 | 0.809 | 0.612 | 2.33E-65 |
| CALCRL | 0.570 | 0.278 | 0.043 | 1.06E-61 |
| CYTIP | 0.698 | 0.475 | 0.330 | 2.65E-61 |
| HPGD | 1.083 | 0.328 | 0.271 | 8.72E-61 |
| PPP1CB | 0.566 | 0.834 | 0.731 | 7.14E-58 |
| AC044893.1 | 0.560 | 0.242 | 0.022 | 1.54E-55 |
| BAALC | 0.596 | 0.723 | 0.415 | 2.22E-55 |
| ABLIM1 | 0.514 | 0.356 | 0.117 | 7.19E-53 |
| TFPI | 0.642 | 0.783 | 0.560 | 1.22E-52 |
| MT-ND2 | 0.550 | 1.000 | 0.998 | 2.76E-51 |
| MT-ND6 | 0.532 | 0.641 | 0.403 | 3.09E-51 |
| SMYD3 | 0.582 | 0.606 | 0.470 | 3.23E-50 |
| PDE4B | 0.764 | 0.654 | 0.415 | 5.17E-47 |
| VCL | 0.510 | 0.609 | 0.408 | 3.45E-46 |
| NPR3 | 0.601 | 0.678 | 0.436 | 2.36E-44 |
| DDX3Y | 0.654 | 0.617 | 0.337 | 2.27E-43 |
| TCF4 | 0.517 | 0.586 | 0.294 | 2.29E-41 |
| S100A4 | 0.634 | 0.990 | 0.931 | 3.15E-41 |
| CDK6 | 0.551 | 0.972 | 0.904 | 3.84E-41 |
| S100A9 | 1.245 | 0.509 | 0.385 | 2.22E-40 |
| HLA-E | 0.516 | 0.959 | 0.895 | 5.29E-35 |
| IFITM3 | 0.566 | 0.761 | 0.518 | 1.73E-34 |
| RGS1 | 0.557 | 0.550 | 0.267 | 6.34E-31 |
| TNFAIP3 | 0.863 | 0.557 | 0.531 | 2.02E-27 |
| NEAT1 | 0.642 | 1.000 | 0.986 | 2.49E-27 |
| RPS4Y1 | 0.549 | 0.761 | 0.492 | 3.78E-27 |
| CCL3L1 | 0.893 | 0.298 | 0.216 | 5.75E-18 |
| KCNQ1OT1 | 0.586 | 0.449 | 0.274 | 8.95E-17 |
| SPINK2 | 0.570 | 0.768 | 0.749 | 1.37E-15 |
| CCL4L2 | 0.917 | 0.111 | 0.045 | 6.01E-08 |
| LSCs: leukemic stem cells; CR: complete remission  *log fold-change of the average expression within LSCs between CR and non-CR patients  **^†^**the percentage of cells that the feature was detected in non-CR patients  **‡**the percentage of cells that the feature was detected in the CR patients | | | | |
