# Supplementary figures and images for: Myeloperoxidase and Thyrotropin‐Releasing Hormone Within Leukaemia Stem Cells Increased Chemosensitivity in Acute Myeloid Leukaemia
Source: J Cell Mol Med. 2024 Dec 25;28(24):e70306. doi: 10.1111/jcmm.70306 (PMC11669111; doi:10.1111/jcmm.70306)

## Supplementary Fig. S1

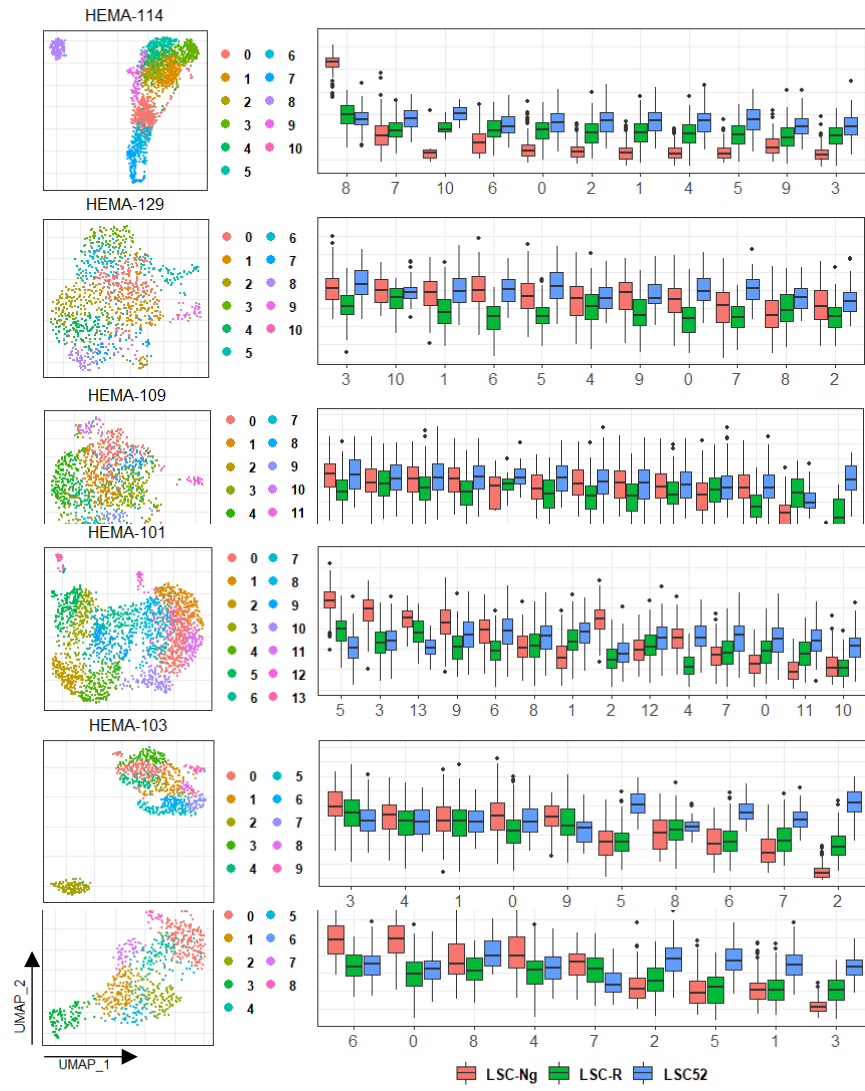

Supplementary Fig. S2

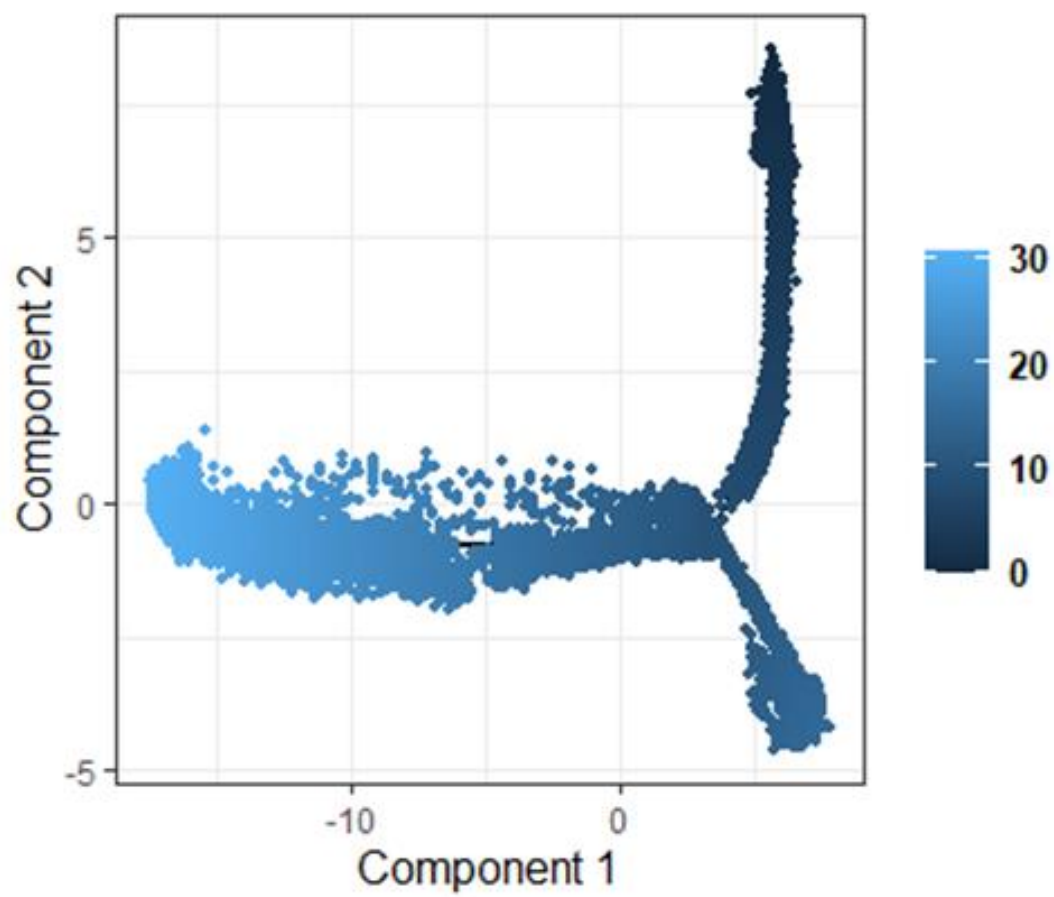

## Supplementary Fig. S3

(A)

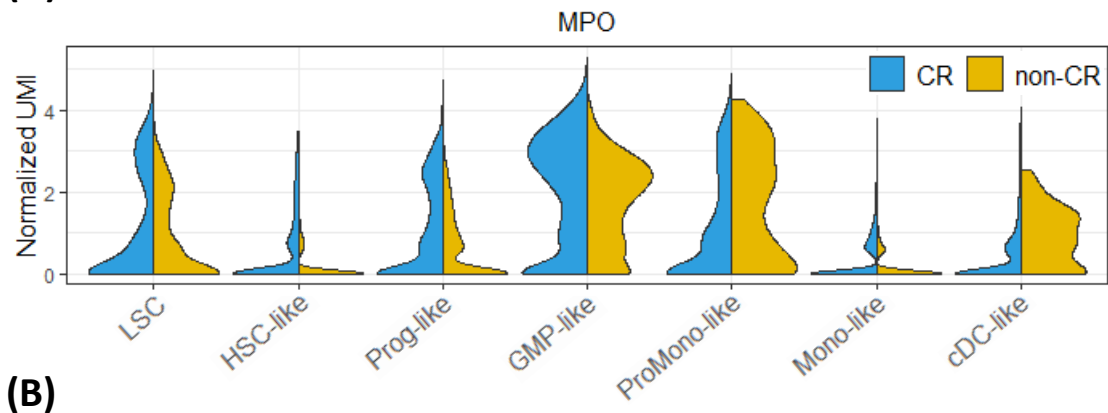

(B)

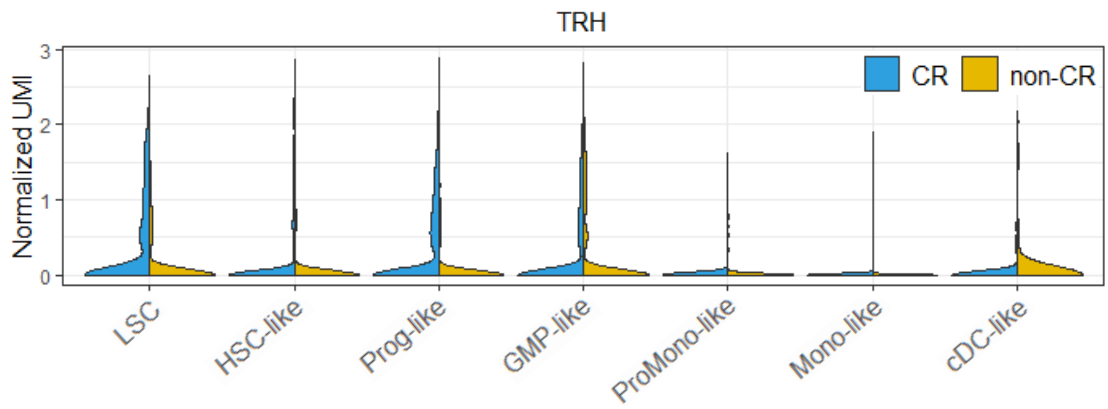

**Supplementary Fig. S4**

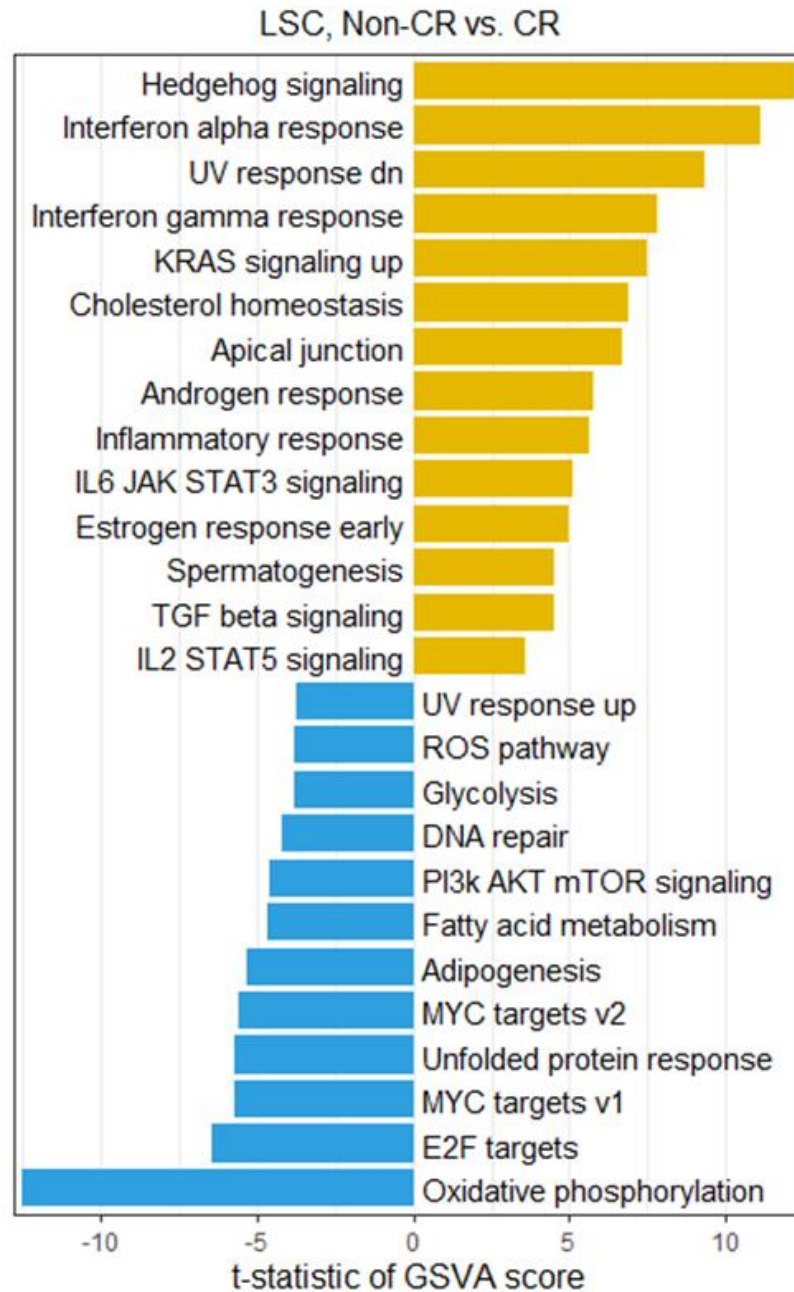

Supplement: Supplementary file 2 — Figures S1–S4.Characterisation of LSCs in a cohort of three patients who achieved CR and three patients who did not attain CR. The top set of samples represents those from patients with CR, while the bottom set pertains to patients without CR. In each sample, the UMAP plot showcases clusters identified through the SNN algorithm, while the boxplot highlights the cluster with the highest stemness levels, determined by calculating GSVA scores from three LSC‐related gene sets: LSC‐Ng, LSC‐R, and LSC52. The clusters within each boxplot are arranged based on the cumulative scores obtained from these assessments. Figure S2 Monocle‐generated plot depicting the differentiation trajectory of malignant cells. The pseudotime was estimated using Monocle, with cells depicted in a lighter colour to indicate a longer pseudotime. Figure S3 the mRNA expression of (A) MPO and (B) TRH between non‐CR and CR patients in malignant cells in each cell type. Figure S4 Comparison of annotated LSCs between patients who did not achieve CR and those who achieved CR. Bar charts illustrate the differential enrichment of hallmark gene sets within LSCs among non‐CR and CR patients. For each gene set, the t‐statistic derived from GSVA scores is reported, and multiple comparisons are adjusted using the Bonferroni correction. Hallmark gene sets with a significance level of 0.05 (Bonferroni‐corrected p‐value < 0.05) are highlighted. The gene sets are organised based on their respective t‐statistics. [file JCMM-28-e70306-s002.pdf]
